# *Aster koraiensis* Nakai: Insights into Its Phytoconstituents and Pharmacological Properties

**DOI:** 10.3390/pharmaceutics18020182

**Published:** 2026-01-30

**Authors:** Anshul Sharma, Hae-Jeung Lee

**Affiliations:** 1 Department of Food and Nutrition, College of Bionanotechnology, Gachon University, Seongnam-si 13120, Republic of Korea; anshulsharma@gachon.ac.kr; 2 Institute for Aging and Clinical Nutrition Research, Gachon University, Seongnam-si 13120, Republic of Korea; 3 Department of Health Sciences and Technology, Gachon Advanced Institute for Health Science and Technology, Gachon University, Incheon 21999, Republic of Korea

**Keywords:** antioxidant, medicinal plant, polyacetylene, inflammation, oxidative stress

## Abstract

**Background/Objectives:***Aster* (*A.*) *koraiensis* (Nakai) Kitamura (synonym *Gymnaster koraiensis*), commonly referred to as Korean starwort, belongs to the composite (Asteraceae) family. This endemic perennial species is cultivated for its long-lasting ornamental flowers and for its young leaves and stems, which serve as a nutritious food source. It grows abundantly across the southern and central regions of the Korean Peninsula, including Jeju Island. The presence of diverse secondary metabolites such as phenolic compounds, polyacetylenes, benzofurans, flavonoids, triperpenoidal saponins, and sesquiterpenoids contributes to its importance in both traditional medicine and modern pharmacology. To date, no comprehensive review has been conducted to summarize its phytoconstituents and pharmacological potential. **Methods:** A non-systematic electronic search of English-language articles using *A. koraiensis* and its synonyms as keywords was conducted to assess its bioactive constituents and health-promoting potential. **Results:** This review seeks to compile and discuss the health-promoting activities of *A. koraiensis*, including its antioxidant, anti-inflammatory, anti-diabetic, anti-tumor, antithrombotic, anticoagulant, anti-angiogenic, antinociceptive, anti-metabolic syndrome, antiviral, hepatoprotective, and cognitive function-enhancing properties, based on evidence from cell and animal studies. To date, more than 75 phytoconstituents have been purified and characterized from this plant. **Conclusions:** The extensive pharmacological activities of *A. koraiensis* highlight its medicinal importance. Future studies should concentrate on the separation, identification, and quantification of its bioactive metabolites, alongside an in-depth investigation of its potential health-enhancing properties.

## 1. Introduction

*Aster* (*A.*) *koraiensis* Nakai (also known as *Gymnaster* (*G.*) *koraiensis*, Beolgaemichwi) is a perennial plant that belongs to the Compositae (Asteraceae) family. It is an indigenous Korean plant and mostly grows in the middle and south of the Korean peninsula, as well as on Jeju Island [1]. Nakai Takenoshin was the first to write about it in 1909 [2,3]. This plant is hermaphroditic, possessing both female and male reproductive parts, and is naturally entomophilous, relying on insects for pollination. It is mostly planted for its ornamental value due to its attractive pale-purple blossoms. The plant is easy to propagate and tolerates environmental stress. Its blossoms are consumed as tea, and the tender shoots are utilized as culinary ingredients, side dishes, and appetizers [4,5]. Historically, the aerial parts and roots of *A. koraiensis* have been used in Korean folk medicine for the treatment of pertussis, antitussive, pneumonia, and chronic bronchitis [6,7,8]. Additional traditional uses recorded in the 17th-century medical text, *Donguibogam* (Principles and Practice of Eastern Medicine), include treatments for venom-induced toxicity, stroke using leaves and stems, wounds using leaves, and sputum-related conditions using roots [9]. *A. koraiensis* leaves have been certified as safe-to-eat material by the Korean Ministry of Food and Drug Safety [10]. The plant usually serves as a ground cover on vacant land because of the expansive growth characteristics of its rhizomes; however, direct planting is also employed for esthetic purposes [11]. *A. koraiensis* is a native plant species primarily sourced from indigenous populations, with limited propagation by low-production wild plant growers [12]. Other species belonging to the genus *Aster* include *Aster tataricus* L.f., *Aster yomena* (Kitam.) Honda, *Aster scabra*, *Aster pseudoglehnii*, and *Aster glehni* Fr. Schm. [13,14,15,16]. Recently, scientific research has increasingly concentrated on the therapeutic potential of medicinal plants, with numerous studies assessing the functional properties of *A. koraiensis* extracts and bioactive constituents using biological model systems. However, to date, no thorough review has been conducted on this plant species. Therefore, this review aims to summarize current knowledge regarding the phytochemical composition and pharmacological properties of *A. koraiensis*.

## 2. Literature Search Strategy

Many electronic databases, including Embase, Google Scholar, and PubMed, were thoroughly explored for research articles on *Aster koraiensis* and *Gymnaster koraiensis,* using various keywords, covering papers up to December 2025. The keywords used included the following: “*Aster koraiensis* or *Gymnaster koraiensis* and health promotion,” “*Aster koraiensis* or *Gymnaster koraiensis* and pharmacological potential,” “*Aster koraiensis* or *Gymnaster koraiensis* and therapeutic potential,” “*Aster koraiensis* or *Gymnaster koraiensis* and phytoconstituents,” “*Aster koraiensis* or *Gymnaster koraiensis* and antioxidant,” “*Aster koraiensis* or *Gymnaster koraiensis* and anti-inflammatory, “*Aster koraiensis* or *Gymnaster koraiensis* and anti-diabetic,” “*Aster koraiensis* or *Gymnaster koraiensis* and anti-non-alcoholic fatty liver disease or hepatoprotective,” “*Aster koraiensis* or *Gymnaster koraiensis* and antinociceptive,” “*Aster koraiensis* or *Gymnaster koraiensis* and anti-tumor,” “*Aster koraiensis* or *Gymnaster koraiensis* and atopic dermatitis,” “*Aster koraiensis* or *Gymnaster koraiensis* and antithrombotic and anticoagulant,” “*Aster koraiensis* or *Gymnaster koraiensis* and anti-metabolic syndrome,” “*Aster koraiensis* or *Gymnaster koraiensis* and antiviral,” “*Aster koraiensis* or *Gymnaster koraiensis* and neuroprotective,” “*Aster koraiensis* or *Gymnaster koraiensis* and cognitive function enhancement.” All biomedical papers published in English were included in this narrative review.

## 3. Biochemical Constituents

Previous studies have reported the presence of various secondary metabolites from *A. koraiensis*, including polyacetylenes (gymnasterkoreayne B and gymnasterkoreayne E) [17,18,19,20,21], triterpenoids [6,22], sesquiterpenoids [8,23], flavonoids [8,23], caffeoylquinic acids [8,10], and benzofurans [8,24]. Hong et al. [25] reported total polyphenol content (mg gallic acid equivalent/mL) and total flavonoid content (mg QE quercetin equivalents/mL) as 2.6 and 336.9, respectively, in *A. koraiensis* ethanol extracts (10 mg/mL) [25]. As summarized in Table 1, more than seventy-five phytoconstituents have been purified and characterized from this medicinal plant.

## 4. Pharmacological Properties

**Figure 1:**
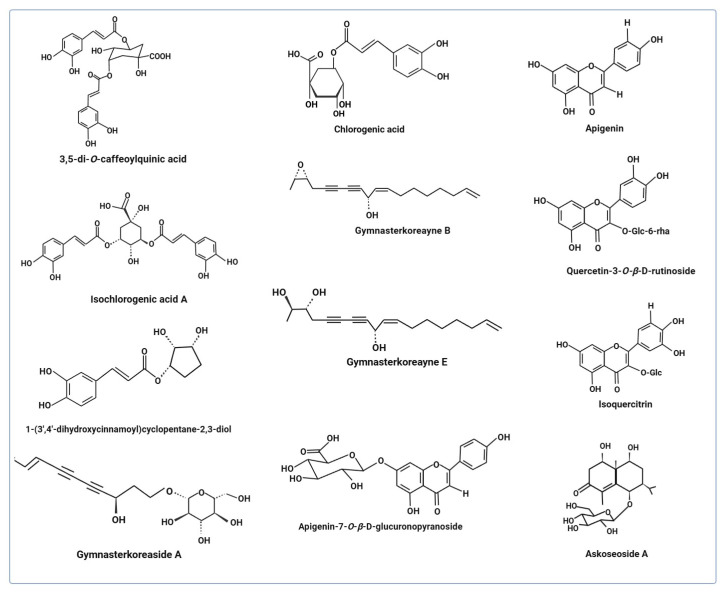
Commonly identified phytochemicals in *A. koraiensis*.

Several studies have reported the health-promoting properties of *A. koraiensis* extracts and the bioactive components derived from its various parts, including the roots, leaves, flowers, and stems. The pharmacological potential of this medicinal plant is primarily attributed to its diverse phytoconstituents. Most available research highlights the beneficial effects demonstrated in various cell-based and animal models. To the best of our knowledge, no clinical data studies have been documented to date. The following review segment summarizes the studies that describe the multiple pharmacological properties of *A. koraiensis*. Figure 1 illustrates the phytochemicals found in *A. koraiensis* that are frequently referenced in later studies.

### 4.1. Antioxidant and Anti-Inflammatory

Medicinal and herbal plants possess a variety of phytoconstituents known to scavenge different ROS. Due to their antioxidant characteristics, researchers are employing these plants to address various ailments through in vitro and in vivo studies [28]. The *A. koraiensis* ethanolic extract showed dose-dependent antioxidant activity for two assays: Trolox equivalent antioxidant capacity and the ferric reducing antioxidant power. Moreover, its scavenging activity against 1,1-diphenyl-2-picrylhydrazyl radical was found to be significantly higher at elevated concentrations. The antioxidant activity of the extract was attributed to the presence of total polyphenol and flavonoid contents in the extract, with greater activity observed at higher extract concentrations (Table 2) [25]. Overall, this study described basic radical-scavenging assays and reducing power tests to exhibit antioxidant activity. The study has several drawbacks, such as the sole use of ethanolic extract and the necessity for relatively high doses to attain maximal activity without identification of specific bioactive compounds. To fully understand the antioxidant potential of this plant, future studies should evaluate different extracts obtained from diverse geographical regions and validate its antioxidant efficacy against oxidative stress using cell and animal models, as well as investigate its effects on stress-related signaling pathways.

Due to the medicinal importance of this plant, continuous research has focused on extracting novel compounds from *A. koraiensis*. One study isolated four new eudesmane-type sesquiterpenoids, three sesquiterpenoids, and two caffeoylquinic acid compounds from the leaf extract of *A. koraiensis* (Table 1) and tested their efficacy in lipopolysaccharide (LPS)-triggered RAW 264.7 cells. Among these compounds, only compound **7**, an eudesmane-type sesquiterpenoid, identified as (1*R*,5*S*,6*R*,7*S*,9*S*,10*R*)-9-*O*-(E-feruloyl)-1,6,9-trihydroxy-eudesm-3-ene-6-*O*-β-D-glucopyranoside*,* demonstrated weak inhibitory effects on the production of prostaglandin E2 and nitric oxide, and the remaining compounds did not show any activity [26] (Table 2). The study suggests the reduced anti-inflammatory activity of the identified compounds, except for compound **7**. Future research should identify bioactive compounds from other parts and assess their pharmacological potential, including anti-inflammatory activity, solely and synergistically, through in vitro and in vivo models.

Dry eye disease is a corneal disease characterized by light sensitivity, itching, and irritation. Inflammation and endoplasmic stress (ER) are believed to play important roles in its pathogenesis. Hong et al. [25] reported that *A. koraiensis* extract mitigates these pathogenic factors in both cell and animal models. In human retinal pigment epithelial cells, treatment with the extract attenuated tumor necrosis factor-alpha (TNF-α)-induced ocular inflammation and thapsigargin-induced ER stress. In animal models, oral administration of the extract reduced tear production, decreased corneal epithelial thickness, increased the inter-tissue gap within the lacrimal glands, and suppressed the activation of the nuclear factor kappa-light-chain-enhancer of activated B cells (NF-kB) [25] (Table 2). The study suggested that 3,5-*O*-dicaffeoylquinic acids and chlorogenic acids might significantly contribute to the reduction in inflammation. Future studies should concentrate on the isolation and characterization of bioactive chemicals from the extract and comprehensively assess their efficacy across various biological functions.

**Figure 2:**
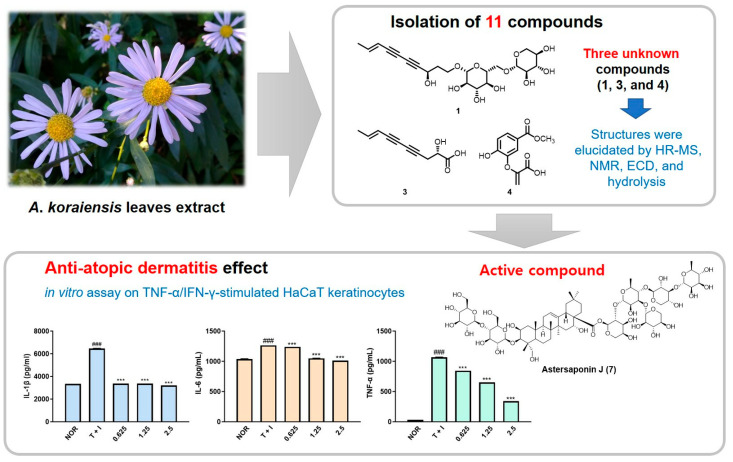
Presentation of the results of evaluating the anti-inflammatory activity of the leaf extract of *A. koraiensis* and eleven isolated compounds, three of which are previously unknown structures identified using various techniques. Among all isolates, superior anti-inflammatory activity was observed for the compound astersaponin J in HaCaT keratinocytes stimulated with TNF-α/IFN-γ, suggesting its potential as a therapeutic candidate for the treatment of atopic dermatitis. All data shown represent the mean ± standard deviation (SD) of triplicate independent experiments. ^###^ *p* < 0.001 vs. the control group; *** *p* < 0.001 vs. TNF-α/IFN-γ-treated group. Adapted from Kim et al. [6]. TNF: tumor necrosis factor; IFN-γ: interferon gamma.

The leaf extract of *A. koraiensis*, along with eleven isolated compounds, namely gymnasterkoreaside A, gymnasterkoreaside C, (2*S*,8*E*)-2-hydroxydeca-8-en-4,6-diynoic acid, gymnasterkoreayne A, spatholosineside A, (2*R*,3*S*)-6-acetyl-2-[1-*O*-(-β-D-glucopyranosyl)-2-propenyl]-5-hydroxy-3-methoxy-2,3-dihydrobenzofuran, dehydrochorismic acid methyl ester, astersaponin J, astersaponin L, astersaponin I, and conyzasaponin J, were evaluated for anti-atopic dermatitis activity in human keratinocyte (HaCaT) cells stimulated with interferon-gamma or TNF-α proinflammatory cytokines. Among the isolated metabolites, compound **1** (gymnasterkoreaside C), compound **3** ((2*S*,8*E*)-2-hydroxydeca-8-en-4,6-diynoic acid), and compound **4** (dehydrochorismic acid methyl ester) were newly identified in this study. Notably, astersaponin J (compound **7**) (at a concentration of 2.5 μM) exhibited the most pronounced anti-inflammatory activity [6] (Figure 2). Additionally, compound **3**, compound **2** (gymnasterkoreaside A), and aliphatic C10 polyacetylenes have been reported for the first time to display anti-atopic actions at a concentration of 10 μM. Future studies should concentrate on the molecular properties of bioactive substances, including astersaponin J, as well as their advantageous effects in animal models.

These studies suggest that different phytoconstituents in the extract of *A. koraiensis* may be responsible for its antioxidant and anti-inflammatory effects. Future research should pay attention to animal studies and safety, as well as to pharmacokinetic studies of the extract and its bioactive compounds. Interactions using the combined effects of bioactive compounds would give us a better idea of the therapeutic potential of identified molecules.

### 4.2. Anti-Diabetic

Diabetes mellitus (DM) is a metabolic disorder that is escalating at an alarming rate, raising global concerns. The global population affected by DM is projected to increase from around 500 million to more than 1 billion by 2050 [29]. Diabetes-associated complications include atherosclerosis, kidney failure, retinopathy, impaired wound-healing ability, peripheral vascular disease, and peripheral sensory neuropathy [30]. Among its two subtypes, the prevalence of type 2 diabetes (T2D) is observed to be higher than that of type 1 [31]. Postprandial hyperglycemia serves as an early clinical marker of T2D, and interventions designed to regulate it may aid in preventing or delaying the advancement of diabetes-related complications. On this line, the blood sugar-lowering effects of *A. koraiensis* leaf extract have been documented in both in vitro and in vivo studies. Extract treatment enhanced glucose transport in 3T3-L1 adipocytes and lowered blood glucose levels in diet-induced obese mice after four weeks of oral administration. The extract also normalized glucose transporter-4 mRNA expression and increased insulin levels, which together contributed to the glucose-lowering effect of the extract (Table 3) [2]. The study is the first to report the glucose-lowering effect of *A. koraiensis* in animal models and enhanced glucose uptake in vitro. However, the study has not evaluated the presence of bioactive compounds, which limits its mechanistic insight and reproducibility.

Medicinal herbs are not only beneficial in managing DM but also in treating secondary conditions, including complications caused by diabetes [32]. Advanced glycation end products (AGEs) are diverse, complex, and sugar-derived irreparable protein alterations that have been linked to diabetes-related complications, such as glomerular podocyte damage and diabetic retinal changes [33].

**Table 3:** The protective effects of *A. koraiensis* extract against diabetes and related complications.

Extract Type	Parts Used	Model and Dose	Major Findings	Ref.
Water	Leaves	3T3-L1 cells + extract (0, 12.5, 25, or 50 μg/mL) + NBDG (150 μg/mL) in glucose-free medium, 1 h	Viability not affected ↑ glucose uptake	[2]
		Diet induced mice + extract (250 or 500 mg/kg), orally (30 min) before glucose (2 g/kg) loading, + metformin (350 mg/kg), 4 weeks	↓ glucose AUC levels: Extract 250, 1002 mg min/dL and extract 500, 1051 mg min/dL, vehicle control group 1313 mg min/dL, ↑ plasma insulin	
Ethanol	Aerial parts	SD rats, streptozotocin (60 mg/kg, i.p.), aminoguanidine (positive control, 100 mg/kg BW), extract (100 and 200 mg/kg BW), 13 weeks	↓ mesangial expansion, proteinuria, and albuminuria. ↓ serum AGEs, ↓ podocytes apoptosis, ↓ Bax, ↑ Bcl-2	[34]
Ethanol	Aerial parts (flowers, stems, and leaves) and roots	Extracted compounds: 3,5-di-*O*-caffeoylquinic acid and CA, In vitro assay	Inhibited AGE formation Inhibited AGE/RAGE binding activity	[5]
		SD rats, normal group, diabetic group, *A. koraiensis* extract-treated group (100 mg/kg BW), once a day, 4 months, STZ (60 mg/kg)	Hyperglycemia or body weight: no significant change, ↓ TUNEL positive cells, ↓ caspase 3 positive cells, ↓ AGEs, ↓ iNOS, ↓ NF-kB	
Ethanol	Flowers, leaves, and stems	SDT rats: Normal group, SDT group, SDT + extract (50 and 100 mg/kg BW), 6 weeks	↓ Blood glucose levels, ↓ BRB breakage, restored occludin, ↓ TUNEL-positive cells, ↓ AGEs	[35]
Ethanol	Flowers, leaves, and stems	Human umbilical vein endothelial cells + extract and CA (0.1, 1, 10 μg/mL) + human VEGF (20 ng/mL), 17 h	Viability not affected Inhibited tube formation CA > extract	[36]
		C57BL/6 + exposure to oxygen 75% on postnatal day 7 (P7) and normal oxygen pressure (P12) + extract (25 and 50 mg/kg BW) + CA (25 and 50 mg/kg BW) per day, last 5 days i.p.	Reduced neovascular tufts size (%) Extract: (25 = 26.27; 50 = 38.75) CA: (25 = 29.68; 50 = 50.24) ↓ VEGF mRNA expression	
Ethanol	Aerial parts	18 compounds *, AGE assay and RLAR inhibition assay	Inhibitory activity against AGEs	[8]
			Inhibition of RLAR	
Ethanol	Aerial parts	Human keratinocytes + extract (3, 10, 30, or 100 μg/mL) + hyperglycemic conditions with glucose (200 mM in media)	Reversed keratinocyte migration: Hyperglycemic cells (13%), control (46%), extract (30 and 100 μg/mL = 21%), ↓ MMP-2, ↓ MMP-9 activity	[37]
		SD rats: normal group, diabetes group, extract (100 mg/kg BW), once daily, 18 days + STZ (75 mg/kg, i.p.)	Faster wound healing in the extract-treated rats compared to the diabetes rats. Reversed skin disruption. ↓ MMP-2, ↓ MMP-9	

* Details have been mentioned in Table 1 and the text of the manuscript. AGEs: advanced glycation end products; RAGE: receptor for AGE; BW: body weight; AUC: area under the curve; BAX: Bcl-2-associated X protein; Bcl-2: B cell lymphoma 2; iNOS: inducible nitric oxide synthase; NF-kB: nuclear factor kappa-light-chain-enhancer of activated B cells; SDT: spontaneously diabetic Torii; i.p.: intraperitoneal; SD: Sprague Dawley; BRB: blood retinal barrier; TUNEL: terminal deoxynucleotidyl transferase dUTP nick end labeling; STZ: streptozotocin; CA: chlorogenic acid; MMP-9: matrix metallopeptidase 9; RLAR: aldose reductase; VEGF: vascular endothelial growth factor; NBDG: 2-[N-(7-nitrobenz-2-oxa-1,3-diazol-4-yl) amino]-2-deoxy-D-glucose. ↓ = decrease, ↑ = increase.

Due to their clinical relevance, there is growing interest in compounds inhibiting AGEs. One study investigated the protective effects of *A. koraiensis* extract derived from plants’ aerial portions on the damage of podocytes in diabetic rats subjected to streptozotocin. Treatment with *A. koraiensis* extract reduced the deposition of AGEs (in a dose-dependent inhibition), prevented podocyte injury, and improved kidney function, including proteinuria and albuminuria. The extract also decreased the expression of B cell lymphoma 2 (Bcl-2) and Bcl2-associated X, apoptosis regulator (Bax) proteins in the renal cortex. These findings imply that *A. koraiensis* extract acts as an inhibitor of AGE deposition and exerts anti-apoptotic effects in the glomeruli of diabetic rats. The presence of caffeoylquinic acids in the extract may contribute to these beneficial effects (Table 3) [34]. Overall, *A. koraiensis* extract shows potential in attenuating the progression of diabetic nephropathy. However, there is no information related to the identification of bioactive compounds from the extract. Additional research is needed to assess the influence of both individual and synergistic actions of bioactive elements, in conjunction with clinical validation.

Similarly, *A. koraiensis* extract (aerial and root parts) demonstrated protective effects by inhibiting AGEs’ accumulation in retinal microvascular cells and suppressing NF-kB activation in streptozotocin-induced rats. High-performance liquid chromatography (HPLC) analysis identified two major compounds in the extract, namely, 3,5-di-*O*-caffeoylquinic acid (22.54 mg/g) and chlorogenic acid (12.35 mg/g) (Figure 1), which inhibited AGEs formation with IC_50_ values of 3.40 and 52.55 μg/mL, respectively, compared to aminoguanidine (IC_50_ = 71.19 μg/mL), a standard glycation inhibitor. Extract treatment (IC_50_ = 84.91 μg/mL) and chlorogenic acid (IC_50_ = 378.78 μg/mL) showed dose-dependent inhibition of AGE/RAGE binding activity, while 3,5-di-*O*-caffeoylquinic acid did not exhibit any effect on receptor binding. Chlorogenic acid inhibited the AGE accumulation, interfered with receptor binding activity, and modulated the downstream signaling pathways. The study suggests that *A. koraiensis* extract and its compounds, particularly chlorogenic acid, hold potential for protecting against injuries of retinal microvascular cells and mitigating progression of diabetic retinopathy (Table 3) [5]. Subsequent research is necessary to assess the retinal protective properties of various compounds, both independently and in synergistic combinations. Furthermore, further pharmacokinetic and safety evaluations are necessary for the extract and its principal bioactive components.

Another study demonstrated the protective effects of *A. koraiensis* extract against diabetic retinopathy in a non-obese model of T2D (spontaneously diabetic Torii rats), which is considered one of the most suitable animal models for studying this condition. The extract suppressed apoptosis and AGEs accumulation in retinal vascular cells as well as inhibited blood retinal barrier breakdown (a clinical feature of diabetic retinopathy) and prevented the loss of tight junction protein, occludin, under diabetic conditions. Occludin plays a critical role in maintaining electrical resistance in retinal vascular endothelium, permeability, and barrier operation (Table 3) [35]. Retinal angiogenesis is commonly associated with retinopathy of prematurity, diabetic retinopathy, and wet age-related macular degeneration. Kim et al. [36] demonstrated the protective effects of *A. koraiensis* extract and chlorogenic acid against retinal neovascularization in an oxygen-induced retinopathy model, wherein animals were exposed to 75% oxygen from postnatal day 7 (P7) to P12 and then returned to normoxic conditions (Table 3). For the next five days (from P12 to P16), animals received treatment either with the extract or compound (each at 25 and 50 mg/kg/body weight per day). Both treatments significantly reduced the neovascular tufts area in animals, with a significantly higher reduction with the chlorogenic acid supplementation (Table 3). Additionally, both the extract and the compound showed dose-dependent inhibition of vascular endothelial growth factor (an angiogenic factor)-stimulated tube formation in human vascular endothelial cells, with chlorogenic acid demonstrating a more potent inhibitory effect. HPLC analysis indicated chlorogenic acid to be the major constituent of the extract. These findings suggest the anti-angiogenic effects of *A. koraiensis* extract are probably due to the presence of chlorogenic acid (Table 3) [36]. Future research should be directed toward safety and pharmacokinetics studies, as well as clinical validation of the extract and the identified compound. All the above-mentioned studies suggest the beneficial effects of *A. koraiensis* extract against diabetic nephropathy and diabetic retinopathy conditions.

In a different study, three new compounds, compound **3** ((2*R*,3*S*)-6-acetyl-2-[1-*O*-(β-d-glucopyranosyl)-2-propenyl]-5-hydroxy-3-methoxy-2,3-dihydrobenzofuran)), compound **1** (9β-*O*-(E-p-hydroxycinnamoyl)-1β,6β-dihydroxy-trans-eudesm-3-en-6-*O*-β-D-glucopyranoside)), and compound **2** (9α-*O*-(E-p-hydroxycinnamoyl)-1α,6α-11-trihydroxy-trans-eudesm-3-en-6-*O*-β-d-glucopyranoside), along with 15 different compounds-3,5-di-*O*-caffeoylquinic acid, 4,5-di-*O*-caffeoylquinic acid, 5-*O*-caffeoylquinic acid, 8*E*-decaene-4,6-diyn-1-*O*-β-D-glucopyranoside, daucosterol, eugenyl-4-*O*-β-D-glucopyranoside, isoquercitrin, isorhamnetin-3-*O*-β-D-glucopyranoside, isorhamnetin-3-*O*-β-D-rutinoside, kaempferol-3-*O*-β-D-rutinoside, larycitrin-3-*O*-α-L-rhamnopyranoside, quercetin-3-*O*-α-L-arabinopyranoside, α-spinasterol, gymnasterkoreayne D, and gymnasterkoreayne B (Table 1), were isolated from ethanolic fraction of aerial parts of *A. koraiensis* extract. Among all, nine compounds were investigated for their inhibitory activity against AGEs and alditol/NADP+ oxidoreductase (RLAR), representing dual bioactivities of compounds against diabetes-related complications. For AGE inhibitions, isoquercitrin (compound **8**), quercetin-3-*O*-α-L-arabinopyranoside (compound **9**), isorhamnetin-3-*O*-β-D-rutinoside (compound **10**), 3,5-di-*O*-caffeoylquinic acid (compound **4**), and 4,5-di-*O*-caffeoylquinic acid (compound **5**) showed inhibitory activity with IC_50_ values of 9.2, 9.0, 8.2, 6.6, and 6.4 μM, respectively. For the RLAR assay, the highest inhibition (IC_50_ = 0.30 μM) was observed for compound **4**, followed by compound **9** and compound **6** (5-*O*-caffeoylquinic acid) with IC_50_ values of 0.32 μM and 0.95 μM, respectively. These findings suggest that compounds belonging to the two major groups, including flavonoids and quinic acids, are credited with the main activities of this medicinal plant. The main benefit of this study is that it measures bioactivity using extracted components instead of just the crude extract. Nevertheless, the study has limitations, such as the lack of in vivo validation, safety, and pharmacokinetic evaluations of the isolated compounds (Table 3) [8].

Delayed wound healing is a well-recognized complication of hyperglycemia in diabetes [38]. In one study, eighteen days of oral supplementation with ethanolic *A. koraiensis* extract improved wound healing by restoring impaired keratinocyte migration, although it did not affect keratinocyte proliferation (Table 3) [37]. Elevated levels of matrix metalloproteinases (MMP-2 and MMP-9) are known to interfere with re-epithelialization during the wound healing process. Treatment with the extract reduced MMP-2/9 activity in keratinocytes as well as their expression in skin tissue. The authors hypothesized that the wound-healing effects of the extract may be attributed to its active constituents, particularly chlorogenic acid. However, scientific validation is required in future research to ascertain that bioactive compounds, notably chlorogenic acid, could improve delayed wound healing conditions. Overall, the study suggests that *A. koraiensis* extract represents a promising anti-diabetic agent with the potential to promote wound healing [37].

Collectively, the studies discussed above indicate the protective potential of *A. koraiensis* extract and its constituents, particularly 3,5-di-*O*-caffeoylquinic acid and chlorogenic acid, against diabetes and related complications. Additionally, pharmacokinetic and safety assessment studies, along with well-designed clinical studies, will be needed in the future.

### 4.3. Anti-Metabolic Syndrome

Worldwide, the prevalence of obesity and metabolic syndrome is rising at a frightening rate. Projections indicate that by 2050, the number of obese individuals worldwide will rise to 3.80 billion [39]. In 2023, the global prevalence of metabolic syndrome rose to approximately 1.54 billion individuals, including 846 million women and 692 million men [40]. Excessive energy intake, coupled with dysregulated energy utilization, significantly contributes to the development of obesity. Modern sedentary lifestyles and physical inactivity are also among the primary causes of this global health concern [41]. As a result, researchers are increasingly investigating natural remedies for the prevention and management of obesity. In this context, Jung et al. [42] evaluated the effects of the ethanolic extract of *G. koraiensis* on metabolic syndrome in a high-fat-fed diet model. Supplemented extract reduced body weight and white adipose tissue (WAT) weight by enhancing energy expenditure. The treatment activated adenosine monophosphate-activated protein kinase (AMPK) signaling, which increased the oxidative capacity of the mitochondria and enhanced lipid catabolism in WAT. The extract also improved insulin resistance, oxidative stress, and inflammation in treated animals (Table 4). The study indicates the multifaceted therapeutic application of *G. koraiensis* extract [42]. Future studies should concentrate on extracts from various solvents, their component identification using diverse methodologies, and their use at varying doses to evaluate compound safety. Comprehensive pharmacokinetic investigations, coupled with clinical validation, are essential to elucidate the impact of this medicinal plant on metabolic syndrome through cellular and animal models.

### 4.4. Hepatoprotective

Extensive oxidative stress is a leading contributor to hepatotoxicity [43]. Numerous studies have emphasized the efficacy of herbal plants and their components in combating liver disorders [44,45]. Among the key chemical compounds present in *A. koraiensis* are polyacetylenes, which possess notable therapeutic potential. For example, gymnasterkoreayne B, a polyacetylene, showed protective effects against menadione (a quinone compound)-induced hepatocyte injury in both cell and animal models (Table 5) [20]. Menadione triggers oxidative stress by generating reactive oxygen species (ROS) [46]. The detoxification of quinones is governed by detoxification enzymes such as quinone reductase (QR), a flavoprotein. Gymnasterkoreayne B treatment protected liver (HepG2) cells from oxidative stress by upregulating erythroid 2-related factor 2 (Nrf2), which induces antioxidant and cytoprotective genes and by enhancing the expression of detoxifying enzymes, NAD(P)H: quinone oxidoreductase (NQO1 or OR, phase II detoxification enzymes) and hemeoxygenase I [20].

This study suggests that gymnasterkoreayne B protects cells from menadione-induced toxicity by inducing two cellular defense systems, detoxification and antioxidation. The findings also support the chemopreventive nature of the compound due to its ability to induce detoxifying enzymes. Gymnasterkoreayne B also elevated the expression of phase I detoxification enzymes; however, this effect was not statistically significant [20]. Overall, the study demonstrates the hepatoprotective effects of gymnasterkoreayne B using both in vitro and in vivo models. However, the study did not report on the safety and pharmacokinetics of the selected compound. Future research should pay attention to further elucidating the protective effects of gymnasterkoreayne B and other compounds from *A. koraiensis*, individually or synergistically, particularly against the hepatotoxic agents.

The ethyl acetate fraction of *G. koraiensis* and its major component, 3,5-di-*O*-caffeoylquinic acid, as well as the hexane fraction of this plant and its constituent, gymnasterkoreayne B, demonstrated protective effects against oxidative stress induced by tert-butyl hydroperoxide (t-BHP) and acetaminophen, respectively, in HepG2 cells (Table 5) [47]. The protective effects of the ethyl acetate fraction and compound were attributed primarily to the radical scavenging activity, increased glutathione levels, and metabolism of t-BHP through a two-electron reduction by the glutathione peroxidase system. In contrast, the hexane fraction and the compound exerted protective effects by inhibiting cytochrome P450 (CYP 3A4), an enzyme responsible for generating hepatotoxic metabolites during oxidation of acetaminophen. Despite the protective effects of hexane extract, its treatment at the highest selected concentration (60 μg/mL) exhibited toxicity in *t*-BHP-induced cells, which could be attributed to the toxic nature of hexane. Similarly, gymnasterkoreayne B reduced cell viability at higher concentration (80 μM). Overall, the study postulates that free radical scavenging against *t*-BHP-induced oxidative stress and inhibition of the CYP 3A4 enzyme, and the induction of detoxification enzymes through activation of the Nrf2 pathway, collectively contributed to the hepatoprotective effects of *G. koraiensis* and its major bioactive compounds (Table 5) [47]. In conclusion, this study exhibited the beneficial effects of ethyl acetate and hexane extracts on oxidative stress in HepG2 cells. Future research should isolate and identify different chemical compounds and evaluate their efficacy in vivo.

Non-alcoholic fatty liver disease (NAFLD) is a prevalent etiology of chronic liver disease worldwide and poses an increasing public health concern [49]. In 2020, it was reclassified as metabolic dysfunction-associated fatty liver disease [50,51]. Researchers are looking for plant-based options for evaluating the protective effects against this liver disease [52,53]. The protective effects of *G. koraiensis* extract have also been reported in a high-fat-fed NAFLD animal model, where the extract reduced hepatic lipid accumulation by inhibiting fatty acid synthesis and cholesterol synthesis. HPLC analysis revealed the presence of isochlorogenic acid A (ICA) as a major component in the extract (Figure 1) [48]. Future research should focus on pharmacokinetics and safety studies of purified compounds from *A. koraiensis*. Evaluations of bioactive compounds solely and synergistically in liver protection and downstream signaling molecules are necessary.

### 4.5. Anti-Tumor

Cancer is a severe health issue that affects people on almost every continent. While advancements in therapeutic treatments have improved survival rates, they often lead to significant negative consequences. Recent research has focused on the beneficial effects of several medicinal plants, including their chemotherapeutic and chemopreventive activities [54]. These natural-product-derived anti-cancer effects are mostly due to the presence of biofunctional elements. The *A. koraiensis* plant is known to contain compounds that exhibit anti-cancer properties (Table 6).

In one study [17], eight compounds (Table 1) were extracted from dried roots of *G. koraiensis* with 80% ethanol and tested for their cytotoxic activity against L1210 tumor cells. Except for compound **1** (gymnasterkoreayne A), all other compounds showed cytotoxicity, with compound **3** (gymnasterkoreayne C) and **8** (2,9,16-heptadecatrien-4,6-diyn-3,8-diol) exhibiting the strongest cytotoxic effects (Table 6). These findings indicate that the presence of the two terminal double bonds in the molecular structure of compounds may play a critical role in enhancing cytotoxicity against mouse leukemia (L1210) tumor cells (Table 6) [17]. The merit of this study resides in the documentation of bioactive compounds; however, the cell viability assessment alone may be interpreted as a limitation. To enhance the anticancer efficacy of isolated compounds, an evaluation of their effects on specific molecular and signaling pathways is essential. Additionally, their effects in non-cancer cell lines, animal models, and well-designed clinical studies are essential.

Aldo-keto reductases (AKR) are a superfamily of NAD(P)H-dependent oxidoreductases, known to function as detoxifying enzymes that metabolize xenobiotic compounds, including drugs and chemical carcinogens. Among these, AKR family 1 B10 (AKR1B10), also referred to as aldose reductase-like-1 (ARL-1), has been documented as a potential serum marker for malignant diseases and is secreted through a lysosomal non-classical pathway [57,58]. AKR1B10 protein is considered a potential therapeutic target, and significant research is focused on identifying its potential inhibitors [59]. A phytoconstituent, 3,5-O-dicaffeoyl-*epi*-quinic acid (3,5-DCQA), extracted with ethyl acetate from the dried aerial part of *G. koraiensis* (Nakai) Kitam, was reported to inhibit AKR1B10 activity. 3,5-DCQA suppressed the activity of AKR1B10 with a 1.2 μM inhibitory concentration (50%). The kinetic parameter estimation using the Lineweaver-Burk linearization illustrated that the inhibition mode was uncompetitive [55]. Overall, the study indicates that the isolated compound exhibits inhibitory activity against the AKR1B10 protein. However, to fully elucidate its anticancer potential, further evaluation in other cancer cell lines and appropriate animal models is required.

Seo et al. [4] identified 22 compounds from *G. koraiensis* extract through chromatographic separation, featuring four newly recognized eudesmane-type sesquiterpene glycosides (askoseosides A, B, C, and D) (Table 1). The extracted compounds were assessed for anti-cancer activity in epidermal growth factor (EGF)-triggered as well as 12-*O*-tetradecanoylphorbol 13-acetate (TPA)-stimulated mouse epithelial cells (JB6 Cl41) (Table 6). Both EGF and TPA are well-known tumor promoters, and the selected in vitro model is widely utilized to elucidate the molecular mode of action of anti-tumor drugs and tumor progression. Among all compounds, the transformation inhibition (% ranged from 22.5 to 88.6% and 36.4 to 80.2%) against EGF- and TPA-induced JB6 Cl41 cells, respectively. Among all identified compounds, apigenin (compound **9**), apigenin-7-O-β-D-glucuronopyranoside (compound **14**), askoseoside D (compound **4**), and 1-(3,4-dihydroxycinnamoyl) cyclopentane-2, 3-diol (compound **22**) exhibited superior activity compared to the other tested compounds [4]. In a nutshell, isolated compounds **9**, **14**, and **22** exhibited superior anticancer activity and did not affect the normal human dermal fibroblast cells’ viability. Future research should investigate both the individual and synergistic effects of these compounds across multiple cancer cell line models. In addition, in vivo validation, along with pharmacokinetic profiling and long-term safety assessments, is warranted.

Polyacetylenes are known to exhibit anti-inflammatory and anti-cancer activities. Based on this concept, GKB or GKB-rich ethanolic extract (GE) was evaluated against inflammation-enhanced colon cancer in vivo (Table 6) [56]. While GKB (500 μmol/kg) exhibited moderate anti-inflammatory activity, the GKB-rich ethanolic extract at the same concentration demonstrated significantly stronger anti-inflammatory and anticancer effects. This enhanced bioactivity was likely attributable to the presence of additional components such as 2,9,16-heptadecatrien-4,6-dyne-8-ol and gymnasterkoreaynes C, B, and E in the GKB-rich ethanolic extract, which may produce synergistic effects not observed with purified GKB alone. Immunohistochemistry analysis demonstrated that GE and GKB (at a high dose) treatment reduced the proliferation of tumor tissue by 16% and 15%, respectively. Future research requires more animal studies for the anticancer and anti-inflammatory potential of other individual purified compounds present in the plant, individually and synergistically [56].

Overall, the above-discussed studies demonstrate the antitumor potential of *G. koraiensis* extracts and isolated compounds. However, in many of the studies presented, compounds were extracted using ethanol; thus, future research should assess the use of different solvents for compound extraction and their antitumor effects. In addition, evaluation against multiple in vitro and in vivo tumor models, along with their safety assessment, is needed.

### 4.6. Antithrombotic and Anticoagulant

Thrombotic disorders have emerged as a global health concern; numerous antithrombotic medications are accessible, although they are not devoid of adverse effects, such as bleeding [60]. Consequently, researchers are exploring alternative and complementary remedies, with herbal medicines seen as the most suitable [61].

A substantial body of preclinical research has documented the antithrombotic, fibrinolytic, and antiplatelet effects of plant-derived extracts and individual phytoconstituents [62,63]. In an interesting study [9], nakaiase (23 kDa), a crude protease, was extracted from the leaf extract of *A. koraiensis* using ethanol precipitation and purified using a fast protein liquid chromatography column. The novel purified nakaiase demonstrated antithrombotic and anticoagulant activities. Compared to the positive control (tissue plasminogen activator), the nakaiase exhibited a stronger antithrombotic effect. The purified protease exhibited amidolytic activity against chromogenic substrates. The minimal activity was recorded for H-D-Phe-Pip-Arg-pNA (1.24560.01 mmoL/min/mg), while the maximum activity was noted for the pyro-Glu-Gly-Arg-pNA (2.36260.02 mmoL/min/mg), followed by N-alpha-benzyloxycarbonyl-D-Arg-Gly-Arg-pNA (2.02060.01 mmoL/min/mg). The action mechanisms of Nakaise were shown by inhibiting the activated factor X and thrombin activities, by synthesizing fibrin and blood clots, and by prolonging the coagulation factors, activating partial thromboplastin time and prothrombin time. Moreover, at a dosage of 20 mg/kg, nakaiase showed minimal hemorrhagic activity while providing substantial protection against thrombin-associated pulmonary thromboembolism in male ICR mice. These findings indicate the pharmacological potential of the extracted enzyme, especially against cardiovascular disease management (Table 7) [9].

The study findings are beneficial, being the sole known research in which a protease enzyme has been extracted from *A. koraiensis* and employed for its anti-thrombosis and anti-coagulant potential. Plant-derived enzymes emerge as eco-friendly and alternative options to microbial and animal enzymes and find applications in the pharmaceutical and biotechnology sectors. However, prior to in vivo application, several limitations should be addressed, including stability concerns and the high cost associated with enzyme purification.

### 4.7. Antinociceptive

The mechanism of pain transmission entails complex interactions between peripheral and central structures, extending from the skin surface to the brain’s cortex. Among several pain types, nociceptive refers to the one that is linked with the stimulation of nociceptors. The use of medicinal plants as pain relievers has received significant attention these days [64]. A study reported the antinociceptive effects of *A. koraiensis* extract using hot-plate paw-licking and tail flick tests, both of which showed increased latency times [65]. The extract also reduced pain-related behavior in the acetic acid-induced writhing test. In the formalin test, the extract showed no effect during the first phase (after 5 min) but significantly reduced nociceptive behaviors during the second phase (beginning 20 min post administration). Similarly, the extract reduced nociceptive responses induced by substance P. To investigate the mechanistic aspects, mice were pretreated with antagonists (intraperitoneally with yohimbine, naloxone, and methysergide) ten minutes prior to extract administration. Yohimbine supplementation attenuated the antinociceptive effect, indicating the protective action was mediated by the alpha 2-adrenergic receptor rather than serotonergic and opioidergic receptors (Table 7) [65]. More in vivo studies, along with safety profiles, are needed in the future for assessing the pain-relieving potential of this plant. Future studies should focus on isolation and identification of multiple compounds from this plant and assess them for their antinociceptive potential.

**Table 7:** Antithrombotic, anticoagulant, and antinociceptive effects of different fractions of *A. koraiensis*.

Extract Type	Parts Used	Model and Dose	Major Findings	Ref.
Ethanol, Nakaiase (protease)	Leaves	In vitro antithrombotic activity: Nakaiase (50, 100, 200, and 500 μg) + alteplase (tPA), positive control (10, 20, and 30 U), 1 h at 37 °C, absorbance at 410 nm Inhibition (%): nakaiase (1, 2, and 4 μg) + thrombin (0.25 U) or FXa (0.5 U), 30 min	Degraded blood clot (at 410 nm), nakaiase (500 μg) = 1.79, tPA (30U) = 1.38 Inhibition (%): thrombin (3.1%, 7.5%, and 11.9%), FXa (9.9%, 13.0%, and 21.0%)	[9]
		Turbidity assay (Fibrin clot inhibition): t-PA (20 U) and nakaiase (30, 20, or 10 μg)	Inhibited fibrin clot formation: by nakaiase: 6.6–82.5%, by tPA: 94.8%	
		In vitro anticoagulant activity Nakaiase (5, 10, 20, and 50 μg) APTT, PT + coagulometer	Nakaiase (at 5, 10, 20, and 50 μg): prolonged APTT by 35.1 s, 54.8 s, 95.2 s, and 112.7 s. Prolonged PT by 14.7 s, 20.1 s, 22.5 s, and 30.5 s	
		ICR mice, Group 1: saline, Group 2: human thrombin (3300 NIHU/mg, tail vein, 0.1 mL), Group 3: thrombin + aspirin (20 mg/kg), Group 4: thrombin + nakaiase (10 mg/kg), Group 5: human thrombin + nakaiase (20 mg/kg), 15 min	Antithrombotic activity Protection (%) of mice: Aspirin-treated (60%) Nakaiase-treated (10 mg/kg = 30%) (20 mg/kg = 50%)	
Ethanol (80%)	*A. koraiensis*	ICR mice + extract (oral administration 200 mg/kg BW) 30 min before Hot-plate paw licking or tail flick tests Acetic acid (1%)-induced writhing and formalin (5%) tests Nociceptive behavioral test (Substance P, 0.7 µg/5 µL intrathecal injection)	↑ Latencies of the tail-flick and hot-plate paw-licking ↓ (70%) writhing numbers. ↓ Nociceptive behaviors following formalin treatment (60% reduction, 2nd phase). Substance P: Cumulative nociceptive response reduced (76%)	[65]

ICR: institute of cancer research; NIHU: national institutes of health unit; APTT: activated partial thromboplastin time; PT: prothrombin time; BW: body weight; tPA: tissue plasminogen activator; FXa: activated factor X; ↓ = decrease, ↑ = increase.

### 4.8. Antiviral

Recently, viral infections have become significant global public health issues, resulting in a notable increase in fatalities and morbidity [66]. Due to the adverse effects accompanying chemical drugs and the rise in resistance, researchers are seeking safe and effective alternatives [67].

A collection of studies has recorded several medicinal plants and their phytoconstituents as antiviral agents [68,69]. The molecular constituents of *A. koraiensis* have also been explored for their antiviral potential. A recent study [27] identified six saponins from the leaf extract of *A. koraiensis* through chromatographic extraction and evaluated their antiviral potential. The isolated compounds included astersaponin I, 3-*O*-β-d-glucopyranosyl-2β,3β,16α,23-tetrahydroxyolean-12-en-28-oic acid 28-*O*-α-L-rhamnopyranosyl-(1→3)-β-d-xylopyranosyl-(1→4)-[β-d-xylopyranosyl-(1→3)]-α-L-rhamnopyranosyl-(1→2)-α-L-arabinopyranoside (compound **5**), and conyzasaponin J, astersaponin J, astersaponin K, and astersaponin L. The study assessed the impending potential of the above-listed compounds against the internalization of the severe acute respiratory syndrome coronavirus 2 (SARS-CoV-2) pseudovirus. SARS-CoV-2 entry follows two different pathways [70]: (1) the endosomal pathway in the angiotensin-converting enzyme 2-positive (ACE2+) human non-small cell lung carcinoma (H1299) cells and (2) the serine protease transmembrane protease serine 2 (TMPRSS2)-facilitated fusion-mediated pathway in ACE2 and TMPRSS2 double-positive (ACE2/TMPRSS2^+^) H1299 cells. Against the first pathway, five compounds (except astersaponin L) exhibited antiviral activity with IC_50_ values below 10 μM. Notably, astersaponin J blocked entry of pSARS-CoV-2 in ACE^2+^ (IC_50_ = 2.92 μM) and ACE2/TMPRSS^2+^ cells (IC_50_ = 2.96 μM) without exhibiting any toxicity. Furthermore, astersaponin J suppressed membrane fusion, thereby blocking both viral entry pathways. However, it did not interfere with the molecular interaction between the SARS-CoV-2 spike and ACE2 receptors. The introduction of Spike-HEK293 cells into a monolayer of ACE2/TMPRSS^2+^ H1299 cells rapidly induced cell-to-cell fusion (Table 8) [27].

In their earlier investigation, the authors examined the inhibitory capacity of astersaponin I derived from the leaves of *A. koraiensis* against the entry mechanisms of SARS-CoV-2 variants (Delta, Alpha, Beta, and Omicron) with comparable effectiveness, additionally impeding syncytium formation. The inhibition of astersaponin I commenced by impeding viral entry at the plasma membrane and within endosomal compartments, primarily through the enhancement of cholesterol levels in the plasma membrane and the disruption of the fusion involving the viral envelope and the membrane of the host cell (Table 8) [16].

These studies indicate the antiviral property of *A. koraiensis* and its extracted compounds. Future studies should focus on other viral and bacterial pathogens. The synergistic antiviral activity of identified compounds should be assessed for future outbreaks.

### 4.9. Neuroprotective and Cognitive Enhancement

Currently, in developing nations, glaucoma is a significant cause of blindness, resulting from structural damage or death of neural cells in the optic nerve, which impairs vision. Historically, glaucoma was seen primarily as a disorder caused by raised intraocular pressure (IOP); however, cases exist in which patients with normal IOP still experience progressive damage to retinal ganglion cells (RGCs) and their axons, which compose the optic nerve [71]. The current understanding recognizes glaucoma as a neurodegenerative disease of the brain and the eye [72]. Overproduction of ROS can lead to the death of RGCs [73].

Kim et al. [74] investigated the protective effects of *G. koraiensis* extract and its isolated compound, 3,5-di-*O*-caffeoylquinic acid (3,5-DCQA), against N-methyl-D-aspartate (NMDA)-induced retinal injury in rats and hydrogen peroxide (H_2_O_2_)-induced oxidative stress in transformed retinal ganglion cells (RGC-5). Both the extract and the compound attenuated H_2_O_2_-induced oxidative stress in RGC-5 cells in a dose-dependent manner and safeguarded the cells from apoptosis-mediated cell death by suppressing the stimulation of pro-apoptotic proteins (Table 9). In animals, NMDA treatment increased ROS generation, leading to neurotoxicity. Both the extract and compound treatments reduced intracellular ROS levels, inhibited lipid peroxidation, increased the thickness of the inner plexiform layer, and reduced the number of terminal deoxynucleotidyl transferase–mediated dUTP nick-end labeling (TUNEL)-positive cells. Moreover, the expression levels of antioxidant proteins, superoxide dismutase, catalase, and glutathione peroxidase-1, were significantly reversed by both the extract and the compound. These results indicate the therapeutic potential of *G. koraiensis* extract and its bioactive component, 3,5-DCQA, in addressing neurodegenerative illnesses, especially glaucoma, where oxidative stress is identified as a primary contributing factor to the disease (Table 9) [74].

The study indicates the pharmacological potential of an active compound, 3,5-DCQA, from this medicinal plant. However, comprehensive safety and pharmacokinetic studies are warranted to support its further development.

Autophagy is a recycling process that breaks down cells and keeps them in balance at both the cellular and organismal levels. The process is needed to maintain the normal physiology of cells by removing damaged organelles, carcinogenic and pathogenic agents, and protein aggregates [75]. Poor regulation of autophagy mediates the development of a variety of human diseases, including neurodegenerative disorders [76].

**Figure 3:**
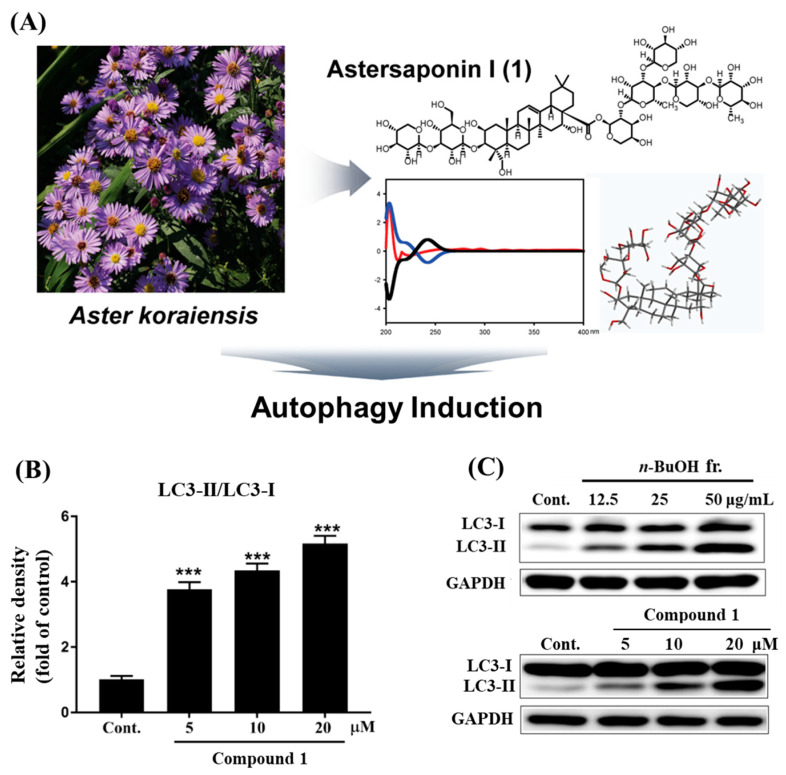
The upper part (**A**) of the figure depicts the isolation of a new triterpene saponin, astersaponin I (compound **1**), and its structural elucidation from the ethanolic extract of *A. koraiensis*. The colored lines represent electronic circular dichroism spectra of compound **1**: red (measured), blue (theoretical), and black (theoretical, enantiomer). The lower half shows autophagy induction by compound **1**. (**B**) The bar graph depicts the up-regulated LC3-II expression in a dose-dependent manner by treatment with compound **1**. (**C**) The representative Western blot bands of protein marker LC3 in SH-SY5Y cells treated with *n*-butanol fraction and compound **1**. Data are expressed as mean ± SEM (*n* = 3). *** *p* < 0.001 represents a significant difference from control. Adapted from Kwon et al. [22] with little modification.

Kwon et al. [22] reported that astersaponin I, extracted from *A. koraiensis*, was evaluated for its autophagy-inducing activity in SH-SY5Y cells. The stimulation of autophagy was validated by the elevated expression of microtubule-associated protein 1A/1B light chain 3 beta (LC3-II) (Figure 3). For compound extraction, dried *A. koraiensis* was ground with ethanol (95%), and the resulting powder extract was further partitioned using different solvents: ethanol, *n*-butanol, *n*-hexane, and ethyl acetate. Among these fractions, at concentrations of 12.5, 25, and 50 μg/mL, *n*-butanol and ethanol showed dose-dependent upregulation of LC3-II, while the remaining two fractions (*n*-hexane (12.5 μg/mL) and ethyl acetate (12.5, 25, and 50 μg/mL)) exhibited weak autophagy-inducing effects. The *n*-butanol fraction was further utilized for the extraction of astersaponin I (Table 9) [22]. Furthermore, the authors noted the study’s limitations, stating that increased LC3-II expression alone is insufficient to confirm autophagy activation. Studies in the absence and presence of an autophagy inhibitor are required (Table 9) [22].

Nonetheless, the neuroprotective effect of astersaponin I against Parkinson’s disease (PD) has been described in a subsequent study using both in vitro and in vivo models. In the 1-methyl-4-phenyl-2,3-dihydropyridium ion-triggered in vitro model, astersaponin I restored the diminished SH-SY5Y cell viability and tyrosine hydroxylase (TH) levels while reducing the elevated α-synuclein level. In the animal PD model, the supplemented compound improved mice’s behavioral performance (latency to fall), and it reestablished dopamine synthesis and TH and α-synuclein expression in the mouse brain tissues. In the animal brain tissue, compound treatment induced autophagy by modulating the expressions of autophagy-related markers and by triggering the extracellular signal–regulated kinases/mammalian target of rapamycin (mTOR) and AMPK/mTOR pathways. These studies indicate that autophagy induction could be a potential therapy for neurodegenerative disorders, particularly against PD (Table 9) [77].

**Table 9:** Neuroprotective and cognitive enhancement properties of different fractions of *A. koraiensis*.

Extract Type	Parts Used	Model and Dose	Major Findings	Ref.
Ethanol (94%)	Aerial parts	RGC-5 cells + pretreatment with EAGK and 3,5-DCQA (10, 1, 0.1, and 0.05 μg/mL) + H_2_O_2_ (300 μM), 24 h	↑ cell viability, ↓ PI and Hoechst 33342 positive cells, ↓ cleaved PARP, ↓ cleaved caspase-3, ↓ apoptosis-inducing factor, ↓ ROS, ↑ rGSH/GSSG ratio, ↑ catalase, ↑ Gpx-1, ↓ Bcl-2	[74]
		SD rats, Group 1: non-treated, Group 2: NMDA (5 nmol, I.I.), Group 3: NMDA + EAGK (2 μg/mL), Group 4: NMDA + 3,5-DCQA (10 nmoL), 7 days. Rat forebrain homogenates + sodium nitroprusside (20 μM)	Inhibited IPL thinning, ↓ TUNEL-positive cells, ↓ Lipid peroxidation, Rat retinas: ↑ SOD, ↑ catalase, ↑ Gpx-1	
Ethanol (95%), butanol, n-hexane, ethylacetate	Dried whole plant	SH-SY5Y cells + ethanol, *n*-butanol, and ethyl acetate extracts (50, 25, and 12.5 μg/mL) + astersaponin I (20, 10, and 5 μg/mL), *n*-hexane extract (12.5 μg/mL)	Ethyl acetate and n-hexane: No effect on LC-3 expression. Ethanol and butanol: ↑ expression of LC-3 II/I dose-dependent. Astersaponin I: ↑ LC-3 II/I expression	[22]
Astersaponin I from *A. koraiensis*	Leaves	SH-SY5Y cells + astersaponin I (5, 10, or 20 µM), 24 h + inhibitor treatment 30 min prior to sample treatment + MPP^+^ (2 mM) 1 h after the compound treatment	↑ LC-3 II/I expression, ↓ sequestosome 1 (p62), ↑ p-Erk, ↑ p-AMPK, ↑ p-ULK, ↓ p-mTOR	[77]
		Autophagy induction: SH-SY5Y + U0126 (10 µM, 30 min) or AMPK (50 nM, 36 h) siRNA + astersaponin I (5 and 10 µM) + MPP^+^ (2 mM) 1 h after the compound treatment + 3-MA (inhibitor)	No significant effect on viability ↑ cell viability, ↑ LC-3 II/I expression, ↓ p62, ↑ TH, ↓ α-synuclein, Activated Erk/mTOR pathway Activated AMPK/mTOR pathway	
		C57BL/6j mice, Group 1 and Group 2 (saline), Group 3 (5 mg/kg ropinirole), Group 4 (5 mg/kg astersaponin I) Group 5 (15 mg/kg astersaponin I (p.o.)) + MPTP (30 mg/kg (i.p.) post 1 h, 8 days	Improved behavior performance Restored dopamine level ↑ TH, ↓ α-synuclein, Induced autophagy	
Ethanol (70%)	Leaves	BV2 microglial + extract (0, 10, 20, 40, and 60 μg/mL)and RAW264.7 cells + extract (0, 5, 10, 12.5, 20, 25, 50, and 100 μg/mL) + LPS (0.5 μg/mL)	BV2 cells: ↑ proliferation Inhibited NO production, RAW264.7: ↓ TNF-α, ↓ COX-2, ↓ iNOS	[10]
		SH-SY5Y cells + Aꞵ (0.1 or 0.3 μM), 21–48 h and extract (0.625 and 1.25 μg/mL)	↓ p-NF-kB, ↓ p-tau protein	
		SD rats, Normal group: diet + saline, Negative control group: diet + scopolamine (S, 1 mg/kg), Low dose group: (103.23 mg/kg/day extract + S), High dose group: (300.51 mg/kg/day extract + S), Donepezil group (positive control): diet + donepezil (1 mg/kg/day) + S., 21 days	↑ Hippocampal ChAT activity ↑ Ach activity, ↑ BDNF, ↑ Bcl-2, ↓ p-ERK/ERK, Y-maze test: ↑ alterations ↑ Latency to escape	
		Memory deficit model: ICR male mice. Normal control: diet + saline, Negative control: diet + scopolamine, Low dose: diet + low dose extract + scopolamine, High dose: diet + high dose extract + scopolamine, Donepezil: diet + donepezil.	Improved long-term and spatial memory	
		Control: transgenic mice (5XFAD) + saline; extract group: mice + extract (OA, 200 mg/kg/day), 21 days	↓ β-amyloid accumulation, Protected neuronal cells	

SD: Sprague Dawley; EAGK: *G. koraiensis* extract; NMDA: N-methyl-D-aspartate; RGC-5: retinal ganglion cells; IPL: inner plexiform layer; TUNEL: terminal deoxynucleotidyl transferase dUTP nick-end labeling; OA: oral administration; SOD: superoxide dismutase; 3,5-DCQA: 3,5-di-*O*-caffeoylquinic acid; PARP: poly(ADP-ribose) polymerase; Gpx-1: glutathione peroxidase-1; H_2_O_2_: hydrogen peroxide; PI: propidium iodide; AIF: apoptosis inducing factor; ROS: reactive oxygen species; rGSH: reduced intracellular glutathione; GSSG: oxidized intracellular glutathione; Bcl-2: B cell lymphoma 2; SH-SY5Y cells: human neuroblastoma cell line; I.I.: intravitreal injection; i.p.: intraperitoneal; LC3: microtubule-associated protein light chain 3; RAW264.7: mouse macrophage cell line; LPS: lipopolysaccharide; NO: nitric oxide; TNF-α: tumor necrosis factor-alpha; COX-2: cyclooxygenase-2; iNOS: inducible nitric oxide synthase; Aβ: amyloid-beta; p-NF-kB: phosphorylated nuclear factor kappa-light-chain-enhancer of activated B cells; p-tau: phosphorylated tau; ChAT: choline acetyltransferase; Ach: acetylcholine; BDNF: brain-derived neurotrophic factor; p-ERK: phosphorylated extracellular signal-regulated kinase; ERK: extracellular signal-regulated kinase; ICR: Institute of Cancer Research; MPTP: 1-methyl-4-phenyl-1,2,3,6-tetrahydropyridine hydrochloride; MPP^+^: 1-methyl-4-phenyl-2,3-dihydropyridium ion; 3-MA: 3-methyladenine; p-AMPK: phosphorylated AMP-activated protein kinase; TH: tyrosine hydroxylase; p-ULK: phosphorylated unc-51-like kinase; p-mTOR: mammalian target of rapamycin. ↓ = decrease, ↑ = increase.

Over 50 million individuals globally are afflicted with Alzheimer’s disease (AD), a progressive neurocognitive disorder [78]. AD is characterized by a gradual deterioration in cognitive abilities and the condition of the neurological system [79]. The accumulation of amyloid-beta plaques, tau phosphorylation, a reduction in the synthesis of acetylcholine (a neurotransmitter), oxidative stress, lipid dysregulation, and the activation of inflammatory pathways are all significant contributory factors [80]. Currently, researchers are focusing on the use of plants and their bioactive compounds as therapeutics for AD [78,81]. The role of inflammation has been closely linked to microglial cells’ deregulation, which can lead to the advancement of AD. In this context, the leaf extract (70% ethyl alcohol) of *A. koraiensis* showed anti-inflammatory effects in LPS-stimulated microglial (BV2) cells and RAW 264.7 macrophages. The extract also demonstrated inhibitory effects on inflammatory protein and production of *p*-tau in amyloid β (1–42)-treated SH-SY5Y cells. N-methyl-D-aspartate (NMDA) receptors, a subtype of glutamate receptors, are recognized for exerting two contrasting actions on the brain. They are crucial for the early stages of memory and learning, yet their hyperactivity may result in neuronal death in AD. The leaf extract exhibited antagonistic effects on the NMDA receptor. In vivo, extract administration improved behavioral details such as the Y-maze, Morris water maze, and passive avoidance, in scopolamine-treated SD rats and ICR mice. Treatment also increased hippocampal choline acetyltransferase and BCL-2 activity, altered hippocampal gene expression (neuroactive ligand–receptor interaction genes), and reduced Aβ accumulation and neuronal cell loss in 5XFAD mutagenic AD model mice. The quantified phenolics using HPLC in the extract, primarily caffeoylquinic acids (chlorogenic acid and isochlorogenic A), are proposed as functional constituents. The study highlights the attenuating effects of *A. koraiensis* in cognitive impairment (Table 9) [10]. Overall, these investigations suggest that the *A. koraiensis* extract and its bioactive constituents may alleviate neurodegenerative disorders by reducing oxidative stress and promoting autophagy. Nevertheless, further animal research and safety and pharmacokinetics studies are necessary to fully evaluate the therapeutic potential of this medicinal plant.

## 5. Conclusions and Future Perspectives

This comprehensive article looks at the pharmacological potential of the extract and numerous phytoconstituents of *A. koraiensis*, popularly known as *G. koraiensis*. Most studies to date have assessed the beneficial properties of extracts obtained using ethanol, ethyl acetate, and hexane from the aerial parts of the plant, including leaves, flowers, and stems, while only a limited number of studies have examined extracts from the roots. Around 79 different phytoconstituents have been purified and identified from this plant, with chlorogenic acid, 3,5-di-*O*-caffeoylquinic acid, astersaponin I, and gymnasterkoreayne B speculated to be primary contributors to the effects observed in cell and animal models. Additionally, the extraction and partial purification of a protease named nakaiase has been reported for antithrombotic activity. From the reviewed literature, it can be deduced that the plant possesses functional characteristics that enable it to combat oxidative stress and inflammation, which are two significant contributors to chronic diseases. Nevertheless, the quantity of research substantiating these advantageous effects in relation to a specific disease remains limited. Therefore, additional research employing various cellular and animal models is necessary to thoroughly understand the potential of this plant. Furthermore, the precise function of the isolated phytoconstituents in connection with disease models is still poorly documented. Future research is necessary to isolate, quantify, and assess the effects of bioactive chemicals both individually and in synergistic mode.

Considering the significant therapeutic potential presented by *A. koraiensis*, it is imperative that future studies should focus on the following:

(1) In-depth examinations of pharmacokinetics and safety profiles employing standardized models are crucial for assessing the actual medicinal potential of this botanical species. Traditionally, the plant was used as a folk remedy for pneumonia, chronic bronchitis, and other illnesses. However, the safety data is essential for comprehending the potential at a translational level. (2) The number of animal studies is comparatively limited, whereas data pertaining to clinical studies are seldom documented. Therefore, in addition to animal studies, meticulously executed, long-term, randomized controlled trials are essential. (3) Future research should concentrate on in-depth mechanistic evaluation of extracts or compounds in animal models. (4) There is an urgent need to investigate the interaction studies between the multiple phytoconstituents of this plant and various drug molecules. Utilizing network pharmacology in conjunction with multi-omics may facilitate the identification of prospective therapeutic candidates from *A. koraiensis*. (5) Despite several health benefits, the standardization of the extract is a critical factor to consider during formulation. The extract preparation method may exhibit batch-to-batch variability, thereby impacting stability and reproducibility. The use of effective tools, such as DNA barcoding and metabarcoding, as well as mass spectrometry, should be used to authenticate, differentiate, and assess the quality of the plant material [82,83,84]. (6) Bioavailability studies for uncommonly documented constituents, such as for this plant, should be interpreted as exhibiting very low bioavailability, as most herbal constituents cause significant concerns due to poor absorption or extensive metabolism of the purified compound [85]. (7) The effect of the extract and bioactive compounds on gut microbiota needs to be investigated in appropriate animal models, as microbiota dysbiosis plays a crucial role in the pathogenesis of metabolic disorders [86]. (8) Research must be undertaken utilizing contemporary technology breakthroughs, including transcriptomics, proteomics, and metabolomics, alongside in vitro propagation and elicitor-based methodologies to reveal the untapped potential of this plant [87].

## Figures and Tables

**Table 1:** Phytoconstituents present in plant parts of *Aster koraiensis*.

Name of the Compound	Identified from	Refs.
(1*R*,5*S*,6*R*,7*S*,9*S*,10*R*)-1,6,9-trihydroxy-eudesm-3-ene-6-*O*-β-d-glucopyranoside	Dried flowers and leaves	[4,26]
(1*R*,5*S*,6*R*,7*S*,9*S*,10*R*)-9-*O*-(E-feruloyl)-1,6,9-trihydroxy-eudesm-3-ene-6-*O*-β-d-glucopyranoside	Leaves	[26]
(1*R*,5*S*,6*R*,7*S*,9*S*,10*R*)-9-*O*-(E-*p*-coumaroyl)-1,6,9-trihydroxy-eudesm-3-ene-6-*O*-β-d-glucopyranoside	Leaves	[26]
(1*R*,5*S*,6*R*,7*S*,9*S*,10*R*)-9-*O*-(Z-*p*-coumaroyl)-1,6,9-trihydroxy-eudesm-3-ene-6-*O*-β-d-glucopyranoside	Leaves	[26]
(1*R*,5*S*,6*R*,7*S*,9*S*,10*S*)-1,6,9-trihydroxy-eudesm-3-ene-1,6-di-*O*-β-d-glucopyranoside	Dried flowers and leaves	[4,26]
(1*R*,5*S*,6*S*,7*R*,9*S*,10*R*)-1,6,9,11-tetrahydroxy-eudesm-3-ene-6-*O*-β-d-glucopyranoside	Dried flowers and leaves	[4,26]
(1*R*,5*S*,6*S*,7*R*,9*S*,10*S*)-1,6,9,11-tetrahydroxy-eudesm-3-ene-1,6-di-*O*—β-d-glucopyranoside	Leaves	[26]
2*R*,3*S*)-6-acetyl-2-[1-*O*-(β-d-glucopyranosyl)-2-propenyl]-5-hydroxy-3-methoxy-2,3-dihydrobenzofuran	Dried plant material and leaves	[6,8]
(2*S*,8*E*)-2-hydroxydeca-8-en-4,6-diynoic acid	Leaf extract	[6]
(3*S,*5*R*,6*R,*7*E,*9*S*)*-*megastigman-7-ene-3,5,6,9-tetrol 3-*O*-β-d-glucopyranoside	Aerial parts	[21]
1-(3,4-dihydroxycinnamoyl)cyclopentane-2,3-diol	Dried flower	[4]
1,9,16-heptadecatriene-4,6-diyn-3,8-diol	Roots, *G. koraiensis*	[17]
1-*O*-syringoyl-β-d-glucopyranoside	Aerial parts	[21]
1β,4β,13-trihydroxy-trans-eudesm-6-ene-1-*O*-β-glucopyranoside	Aerial parts and dried flowers	[21]
1β,6β,9α,11-tetrahydroxy-trans-eudesm-3-ene-6-*O*-β-d-glucopyranoside	Leaves	[4,26]
2(*E*),9(*Z*),16-heptadecatriene-4,6-diyn-8-ol	Dried flower	[4]
2,9,16-heptadecatrien-4,6-diyn-8-ol	Roots, *G. koraiensis*	[17]
3,5-di-*O*-caffeoylquinic acid	Aerial parts (flowers, leaves, and stems), dried plant material, and leaves	[5,8,26]
3,8-dihydroxydec-9-en-4,6-yne-1-*O*-β-d-glucopyranoside	Aerial parts	[21]
3-*O*-β-D-glucopyranosyl-2*β*,3*β*,16*α*,23-tetrahydroxyolean-12-en-28-oic acid 28-*O*-α-L-rhamnopyranosyl-(1→3)-β-d-xylopyranosyl-(1→4)-[β-d-xylopyranosyl-(1→3)]-α-L-rhamnopyranosyl-(1→2)-α-L-arabinopyranoside	Leaf extract	[6]
4,5-di-*O*-caffeoylquinic acid	Dried plant material and leaves	[8,26]
4-*O*-caffeoylquinic acid	Leaves	[10]
5-*O*-caffeoylquinic acid	Dried plant material	[8]
6″-*O*-(syringoyl)-1β,6β,9β,11-tetrahydroxy-trans-eudesm-3-en-6-*O*-β-d-glucopyranoside	Aerial parts	[21]
8*E*-decaene-4,6-diyn-1-*O*-β-d-glucopyranoside	Dried plant material	[8]
9α-*O*-(E-p-hydroxycinnamoyl)-1α,6α-11-trihydroxy-trans-eudesm-3-en-6-*O*-β-d-glucopyranoside	Dried plant material, aerial parts	[8,21]
9β-*O*-(E-p-hydroxycinnamoyl)-1β,6β-dihydroxy-trans-eudesm-3-en-6-*O*-β-d-glucopyranoside	Dried plant material, aerial parts	[8,21]
Alangioside A	Aerial parts	[21]
Apigenin	Dried flower	[4]
Apigenin-7-*O*-β-d-glucuronide methyl ester	Dried flower	[4]
Apigenin-7-*O*-β-d-glucuronopyranoside	Dried flower	[4]
Apigenin-7-*O*-glucoside	Leaves	[10]
Askoseoside A	Dried flower	[4]
Askoseoside B	Dried flower	[4]
Askoseoside C	Dried flower	[4]
Askoseoside D	Dried flower	[4]
Astersaponin I	Extract, whole plant, and leaves	[6,22,27]
Astersaponin J	Leaves	[6,27]
Astersaponin K	Leaf extract	[27]
Astersaponin L	Leaf extract	[6,27]
Cannabiside D	Aerial parts	[21]
Carpeside B	Aerial parts	[21]
Chlorogenic acid	Aerial parts (flowers, leaves, and stems)	[5]
Citrusin C	Aerial parts	[21]
Conyzasaponin J	Leaf extract	[6,27]
Daucosterol	Dried plant material	[8]
Dehydrochorismic acid methyl ester	Leaf extract	[6]
Eugenol rutinoside	Aerial parts	[21]
Eugenyl-4-*O*-β-d-glucopyranoside	Dried plant material	[8]
Gymnasterkoreaside A	Aerial parts, dried plant material, leaves, and roots of *G. koraiensis*	[6,8,18,21]
Gymnasterkoreaside B	Roots of *G. koraiensis*	[18]
Gymnasterkoreaside C	Leaf extract	[6]
Gymnasterkoreayne A	Leaf extract and roots of *G. koraiensis*	[6,17]
Gymnasterkoreayne B	Aerial parts, dried plant material, roots of *G. koraiensis*, and dried flowers	[4,8,17,21]
Gymnasterkoreayne C	Dried flowers and roots of *G. koraiensis*	[4,17]
Gymnasterkoreayne D	Aerial parts, dried plant material, and roots of *G. koraiensis*	[8,17,21]
Gymnasterkoreayne E	Dried flowers, aerial parts, dried plant material, and roots of *G. koraiensis*	[4,17,21]
Gymnasterkoreayne F	Roots of *G. koraiensis*	[17]
Gymnasterkoreayne G	Dried flower	[4]
Isochaftoside	Leaves	[10]
Isochlorogenic acid A	Leaves	[10]
Isochlorogenic acid B	Leaves	[10]
Isochlorogenic acid C	Leaves	[10]
Isoquercitrin	Dried plant material, aerial parts	[8,21]
Isorhamnetin-3-*O*-β-d-glucopyranoside	Dried plant material, aerial parts, and dried flowers	[4,8]
Isorhamnetin-3-*O*-β-d-rutinoside	Dried plant material and dried flowers	[4,8]
Kaempferol-3-*O*-β-d-rutinoside	Dried plant material	[8]
Larycitrin-3-*O*-α-L-rhamnopyranoside	Dried plant material	[8]
Linarin	Aerial parts	[21]
Luteolin	Leaves	[10]
Luteolin 7-*O*-glucoside	Leaves	[10]
Neochlorogenic acid	Leaves	[10]
Phlomisiomoside	Aerial parts	[21]
Quercetin-3-*O*-β-d-glucopyranoside	Dried flower	[4]
Quercetin-3-*O*-β-d-rutinoside	Dried flower	[4]
Quercetin-3-*O*-α-L-arabinopyranoside	Dried plant material	[8]
Scopolin	Aerial parts	[21]
Spatholosineside A	Aerial parts, leaf extract	[6,21]
α-spinasterol	Dried plant material	[8]

**Table 2:** Antioxidant and anti-inflammatory effects of *A. koraiensis*.

Extract Type	Parts Used	Assay, Model, and Dose	Major Findings	Ref.
Ethanol	Leaves and flowers	Extract (10, 5, 1, 0.5, and 0.1 mg/mL) TEAC	Scavenging (g TE/mL): 10 = 2.9, 5 = 2.7, 1 = 0.9, 0.5 = 0.4, 0.1 = 0.2	[25]
		Extract (10, 5, 1, 0.5, and 0.1 mg/mL) FRAP	Scavenging (nM FeSO_4_/mL): 10 = 9.7, 5 = 7.8, 1 = 2.0, 0.5 = 1.0, 0.1 = 0.2	
		Extract (10, 5, 1, 0.5, and 0.1 mg/mL) DPPH	Scavenging (mg ascorbic acid/g): 10 = 118.4, 5 = 117.8, 1 = 112.7, 0.5 = 80.0, 0.1 = 30.8	
Ethanol (95%) #	Dried leaves	RAW 264.7 cells + LPS (1 μg/mL, 1 h) + compounds **1**–**9** (100 μM), 24 h	Compound 7 IC_50_ (μM) NO = 95.7and PGE2 = 111.6 Other compounds inactive	[26]
Ethanol	Leaves and flowers	ARPE-19 cells + TNF-α (10 μg/mL) or thapsigargin (5 μmol/L)	TNF-α: ↓ IL-1β, ↓ TNF-α, ↓ IL-8, ↓ IL-6, ↓ MMP-9, ↓ P-p-38, ↓ P-p-ERK. Thapsigargin: ↓ VEGF-α, ↓ calcium ion efflux.	[25]
		BALB/c mice, scopolamine (200 μL, i.p., twice) daily + *A. koraiensis* extract (AKE) (100, 50, or 10 mg/kg), once/day, 2 weeks	Ameliorated corneal damage, ↑ tear production (dose-dependent), reversed TBUT (100 mg/kg), and inhibited thinning of corneal epithelium. Corneal tissue and lacrimal glands: ↓ IL-1β, ↓ IFN-γ, ↓ TNF-α, ↓ MMP-9 Lacrimal gland: ↓ p-IkB/ IkB, ↓ p-NF-kB/NF-kB	
Ethanol (95%) *	Leaves	HaCaT keratinocytes + extract (125, 250, and 500 μM) and compounds (0.625, 1.25, 5, and 10 μM, 1 h) before treatment with TNF-α or IFN-γ (10 ng/mL), 24 h	↓ IL-1 β, ↓ TNF-α, ↓ IL-6	[6]

* Eleven different compounds were isolated; check the text for relevant details. # Nine different compounds were isolated. IC_50_: Half maximal inhibitory concentration; DPPH: 1,1-diphenyl-2-picrylhydrazyl; FRAP: ferric reducing antioxidant power; TEAC: Trolox equivalent antioxidant capacity; TE: Trolox equivalent; LPS: lipopolysaccharide; NO: nitric oxide; PGE2: prostaglandin E2; ARPE-19: human retinal pigmented epithelial-19; i.p.: intraperitoneal; TBUT: tear breakup time; TNF-α: tumor necrosis factor-alpha; IL-1β: interleukin 1 beta; IFN-γ: interferon gamma; MMP-9: matrix metallopeptidase 9; NF-kB: nuclear factor kappa B; p-ERK: phospho-extracellular signal regulated kinase; VEGF-α: vascular endothelial growth factor-alpha. ↓ = decrease, ↑ = increase.

**Table 4:** The protective effects of *G. koraiensis* extract against metabolic syndrome.

Extract Type	Parts Used	Model and Dose	Major Findings	Ref.
Ethanol	Foliage part	3T3-L1 cells + extract (10, 20, 40, and 80 μg/mL) + IBMX 0.1% (0.5 mM), + DEX 0.05% (2.5 mM), insulin 0.1% (10 mg/mL)	↓ lipid accumulation in cells, ↓ C/EBPα, ↑ p-ACC/ACC, ↓ SREBP-1, ↓ FAS, ↓ TG in media	[42]
		C57BL/6N mice, normal group, HFD group, HFD + Orlistat (30 mg/kg), HFD + extract (100 mg/kg), oral administration, 8 weeks	↓ body weight, ↓ epididymal, perirenal, and mesenteric WAT. In serum: ↓ TG, ↓ TC, ↓ LDL-C, ↓ leptin, ↓ ghrelin, ↑ adiponectin. Epididymal WAT: ↓ size and number of adipocytes, ↓ PPAR-γ, ↓ C/EBPα, ↓ SREBP-1, ↓ FAS, ↑ p-AMPK/AMPK, ↑ p-ACC/ACC, ↑ CPT1. ↓ MDA, ↑ NQO1, ↑ HO-1, ↑ SOD2, ↓ serum IL-1β. WAT: ↓ IL-1β, ↓ IFNγ, ↓ M1-specific markers. Improved fasting glucose. Energy expenditure: ↑ p-AMPK, ↑ Cyto C, ↑ PRDM16, ↓ UCP1, ↑ PPARα, ↑ PGC1α, ↑ Nrf2, ↑ TFAM, ↑ CPT2, ↑ UCP3, ↑ ATGL, ↑ HSL, ↑ PEPCK, ↓ PDK4.	

IBMX: 3-isobutyl-1-methylxanthine; HFD: high-fat diet; DEX: dexamethasone; TG: triglyceride; TC: total cholesterol; LDL-C: low- density lipoprotein cholesterol; WAT: white adipose tissue; PPAR-γ: peroxisome proliferator-activated receptor gamma; C/EBPα: CCAAT enhancer-binding protein alpha; SREBP-1: sterol regulatory element-binding transcription factor 1; FAS: fatty acid synthase; p-AMPK: phosphorylated adenosine monophosphate-activated protein kinase; p-ACC: phosphorylated acetyl-CoA carboxylase; CPT1: carnitine palmitoyltransferase-1; MDA: malondialdehyde; NQO1: NAD(P)H quinone dehydrogenase 1; HO-1: heme oxygenase 1; SOD2: superoxide dismutase 2; IL-1β: interleukin-1 beta; IFNγ: interferon gamma; Cyto C: cytochrome C; PRDM16: PR domain containing 16; PPARα: peroxisome proliferator-activated receptor alpha; PGC1α: peroxisome proliferator-activated receptor gamma coactivator alpha; Nrf2: nuclear factor erythroid 2-related factor 2; TFAM: mitochondrial transcription factor A; CPT2: carnitine palmitoyltransferase 2; UCP1/3: uncoupling protein 1/3; ATGL: adipose triglyceride lipase; HSL: hormone-sensitive lipase; PEPCK: phosphoenolpyruvate carboxykinase; PDK4: pyruvate dehydrogenase kinase 4. ↓ = decrease, ↑ = increase.

**Table 5:** The hepatoprotective effects of *A. koraiensis* extract.

Extract Type	Parts Used	Model and Dose	Major Findings	Ref.
Ethanol (94%)	Aerial parts	Hepa1c1c7 cells (0–125 μM, GKB), HepG2 cells (50 μM GKB, 24 h), for GSH, pretreatment with 10 μM GKB, 24 h + 40 μM menadione, 3 h, cell viability (pretreated with GKB 0.8, 1.6, 3.1, 6.3, 12.5 μM), 24 h + menadione (40 μM), 3 h	↑ QR in Hepa1c1c7 cells HepG2 cells: ↑ QR, ↑ HO-1, ↑ GSR, ↑ Nrf2 and its nuclear translocation, ↑ GSH Protection against menadione induced cytotoxicity	[20]
		Certified Disease-Free Fisher 344 (CDF 344) rats; control, sulforaphane (500 μmol/kg BW); GKB (1000 and 500 μmol/kg of BW), 5 days	↑ QR activity in liver. Phase II detoxification enzymes: ↑ NQO1 (QR), ↑ Gsta2, ↑ Ugt1a6, ↑ Gsta3, ↑ Ugt2b17, ↑ Ugt2a1, ↑ GSR	
EA	--	HepG2 cells + *t*-BHP (200 μM) + extract, EA extract, hexane extract (7.5, 15, 30, and 60 μM), DCQA (1.25, 2.5, 5, 10, 20, 40, 80), 24 h	↑ cell viability, ↑ GSH, Reduced DNA damage, ↓ apoptosis	[47]
Hexane		HepG2 cells + acetaminophen (40 mM) + extract, EA extract, hexane extract (3.5, 7.5, 15, 30, and 60 μM), GKB (1.25, 2.5, 5, 10, 20, 40, 80), 24 h	↑ cell viability, ↑ GSH, ↓ sub G0/G1 content, ↓ CYP 3A4 activity, ↓ apoptosis	
Ethanol (95%)	Flowers	C57/BL6J + normal group, HF group, HF + silymarin (200 mg/kg), HF + extract (125, 250, and 500 mg/kg), 12 weeks	↓ body weight gain (dose-dependent), ↓ liver fat, ↓ epididymal fat, ↓ mesenteric fat, ↓ ALT, ↓ AST, ↓ TG, ↓ TC, ↓ LDL-C, ↓ NAFLD activity score, ↓ lobular inflammation score, ↓ ballooning score, ↓ subsection steatosis score	[48]

BW: body weight; GKB: gymnasterkoreayne B; QR: quinone reductase; NQO1: NAD(P)H dehydrogenase quinone 1; Gsta2: Glutathione-S-transferase alpha type2; Ugt1a6: UDP-glycosyltransferase 1 family, polypeptide A6; Gsta3: Glutathione-S-transferase type A3; Ugt2b17: UDP-glucuronosyltransferase 2 family, polypeptide B17; Ugt2a1: UDP-glucuronosyltransferase 2 family, polypeptide A1; GSR: glutathione reductase; HO-1: hemeoxygenase 1; Nrf2: nuclear factor erythroid 2-related factor 2; GSH: glutathione; t-BHP: tert-butyl hydroperoxide; HF: high fat; DCQA: 3,5-di-*O*-caffeoylquinic acid; EA: ethylacetate; TG: triglyceride; TC: total cholesterol; LDL-C: low-density lipoprotein cholesterol; ALT: alanine aminotransferase; AST: aspartate aminotransferase; NAFLD: non-alcoholic fatty liver disease. ↓ = decrease, ↑ = increase.

**Table 6:** Anti-tumor properties of different fractions of *A. koraiensis*.

Extract Type	Parts Used	Model and Dose	Major Findings	Ref.
Ethanolic	Dried roots	Mouse leukemia L1210 cells, compounds treatment (1–8) ^$^, 48 h	ED_50_ (μg/mL) value: C3 (2.1) and C8 (0.12). C2 (3.3), C4 (7.7), C5 (9.6), C6 (3.1), C7 (10.4), Cisplatin (0.02)	[17]
Ethyl acetate	Dried arial parts	Recombinant human AKR1B10	3,5-O-dicaffeoyl-epi-quinic acid inhibited AKR1B10	[55]
Ethanol (70%)	Dried flower	JB6 Cl41 cell + EGF or TPA (10 ng/mL) + each compound (50 μM) *, two weeks	Inhibited cell transformation	[4]
		NHDF + compounds **9**, **14**, and **22** ** (6.25, 12.5, 25, and 50 μM), 48 h	No significant effect on cell viability	
Ethanol	Dried plant material	C57BL/6 mice, GKB or GKB-rich EE = GE (500 or 250 μmol/kg diet daily) + AOM (10 mg/kg) i.p. day 0 and DSS 7–13 days	↑ NQO1 = GE > GKB, ↓ DAI score = GE > GKB, ↓ adenocarcinomas multiplicity 90% high dose GE, ↓ COX-2 in tumor tissue, ↓ IL-6 by GE (both doses)	[56]

^$^ Compounds (**C1**–**C6**): gymnasterkoreaynes A−F; C7: 2,9,16-heptadecatrien-4,6-diyn-8-ol; C8: 1,9,16-heptadecatriene-4,6-diyn-3,8-diol. * 22 compounds, ** compound **9**: apigenin, compound **14**: apigenin-7-*O*-β-D-glucuronopyranoside, compound **22**: 1-(3,4-dihydroxycinnamoyl) cyclopentane-2,3-diol. ED_50_: effective dose to inhibit the concentration by 50%; AKR1B10: aldo-keto reductase family 1 member B10; JB6 Cl41 cell: mouse epidermal cell line; EGF: epidermal growth factor; TPA: 12-O-tetradecanoylphorbol 13-acetate; GKB: polyacetylene gymnasterkoreayne B; EE: ethanol extract; NHDF: normal human dermal fibroblast; AOM: azoxymethane; DSS: dextran sodium sulfate; NQO1: nicotinamide adenine dinucleotide (phosphate) hydride-quinone oxidoreductase 1; DAI: disease activity index scores; COX-2: cyclooxygenase-2; IL-6: interleukin-6; ↓ = decrease, ↑ = increase.

**Table 8:** Antiviral properties of *A. koraiensis*.

Extract Type	Parts Used	Model and Dose	Major Findings	Ref.
Ethanol (95%)	Flowers	ACE2-positive (ACE2^+^) cells	IC_50_ values: astersaponin I = 1.46 μM, astersaponin J = 2.92 μM, astersaponin K = 3.16 μM, astersaponin L = no activity, compound **5** = 6.19 μM, conyzasaponin J = 7.26 μM	[27]
		ACE2/TMPRSS2^+^ H1299 cells	IC_50_ value: astersaponin J = 2.96 μM	
Ethanol (95%)	Leaves	ACE2^+^ cells	IC_50_ values: extract = 48.4 μg/mL, astersaponin I =1.60 μM	[16]
		ACE2/TMPRSS2^+^	IC_50_ values: extract = 62.4 μg/mL, astersaponin I = 2.89 μM	

ACE2: angiotensin-converting enzyme 2; TMPRSS2: the serine protease transmembrane protease serine 2; H1299: human non-small cell lung cancer cell line; IC_50_: extract concentration to inhibit population by 50%.

## Data Availability

No new data were created or analyzed in this study.
